# A rare case of prostate neuroendocrine tumor: A case report

**DOI:** 10.3389/fonc.2022.1009146

**Published:** 2022-10-03

**Authors:** Shunxing Teh, Fam Xeng Inn, Iqbal Hussain Rizuana, Wan Muhaizan WM

**Affiliations:** ^1^ Department of Surgery, Universiti Kebangsaan Malaysia Medical Center, Kuala Lumpur, Malaysia; ^2^ Urology Unit, Department of Surgery, Universiti Kebangsaan Malaysia Medical Center, Kuala Lumpur, Malaysia; ^3^ Department of Radiology, Universiti Kebangsaan Malaysia Medical Center, Kuala Lumpur, Malaysia; ^4^ Department of Pathology, Sunway Medical Center, Sunway, Malaysia

**Keywords:** prostate neuroendocrine tumor, small cell carcinoma, immunohistochemical markers, synaptophysin, chromogranin A

## Abstract

Small cell prostate neuroendocrine carcinoma (SCPC) is a rare and highly aggressive malignant tumor. We present a case of a 52-year-old Iranian man, presenting with complaints of occasional gross hematuria and perineal pain for 6 months. PSA was 0.8 ng/ml. A digital rectal examination found a huge and hard prostate mass. He underwent a transrectal ultrasound-guided (TRUS) biopsy of the prostate. Histopathology showed high-grade small cell neuroendocrine carcinoma. Immunohistochemical markers were positive for synaptophysin with a Ki67 index of almost 100%. However, CD56 and chromogranin A markers were negative. Magnetic resonance imaging (MRI) of the prostate showed a prostate mass with invasion to the rectum, while contrast-enhanced computed tomography of the thorax, abdomen, and pelvis (CT TAP) ruled out metastasis. A multidisciplinary team discussion was carried out, and a decision was made for concurrent chemotherapy and radiation (cisplatin and etoposide for 4 cycles and 70 Gy, 35 fractions). There is a lack of consensus on the management of SCPC. The main modality of management in advanced (stage IV) disease is chemotherapy. It is a highly aggressive tumor with a poor prognosis and is not responsive to hormonal therapy.

## Introduction

Prostate carcinoma is the most common non-cutaneous malignancy, accounting for the second highest cancer-related mortality among men in Western countries (1, 2). According to the Malaysia National Cancer Registry Report from 2012 to 2016, it was the seventh most common cancer among all populations (3.6%) and the third most common cancer among men (7.7%) (3–6). The most common type of prostate carcinoma is adenocarcinoma, and it commonly develops in the glandular region (7). Neuroendocrine cells represent as part of normal prostatic tissue, usually found within periurethral and ductal regions (8, 9). Neuroendocrine prostate carcinoma encompasses a disease spectrum, evolving from prostate adenocarcinoma to small cell carcinoma, either in pure or mixed forms. Primary small cell prostate carcinoma (SCPC) is a very rare entity, which may develop *de novo*, while a more common form may develop in later stages of adenocarcinoma after receiving hormonal therapy (9–12). We are presenting a rare case of a locally advanced (cT4) pure small cell prostate cancer. It is extremely rare to find a localized advanced disease without distant metastasis due to the aggressiveness of the disease.

## Case report

**Figure 1:**
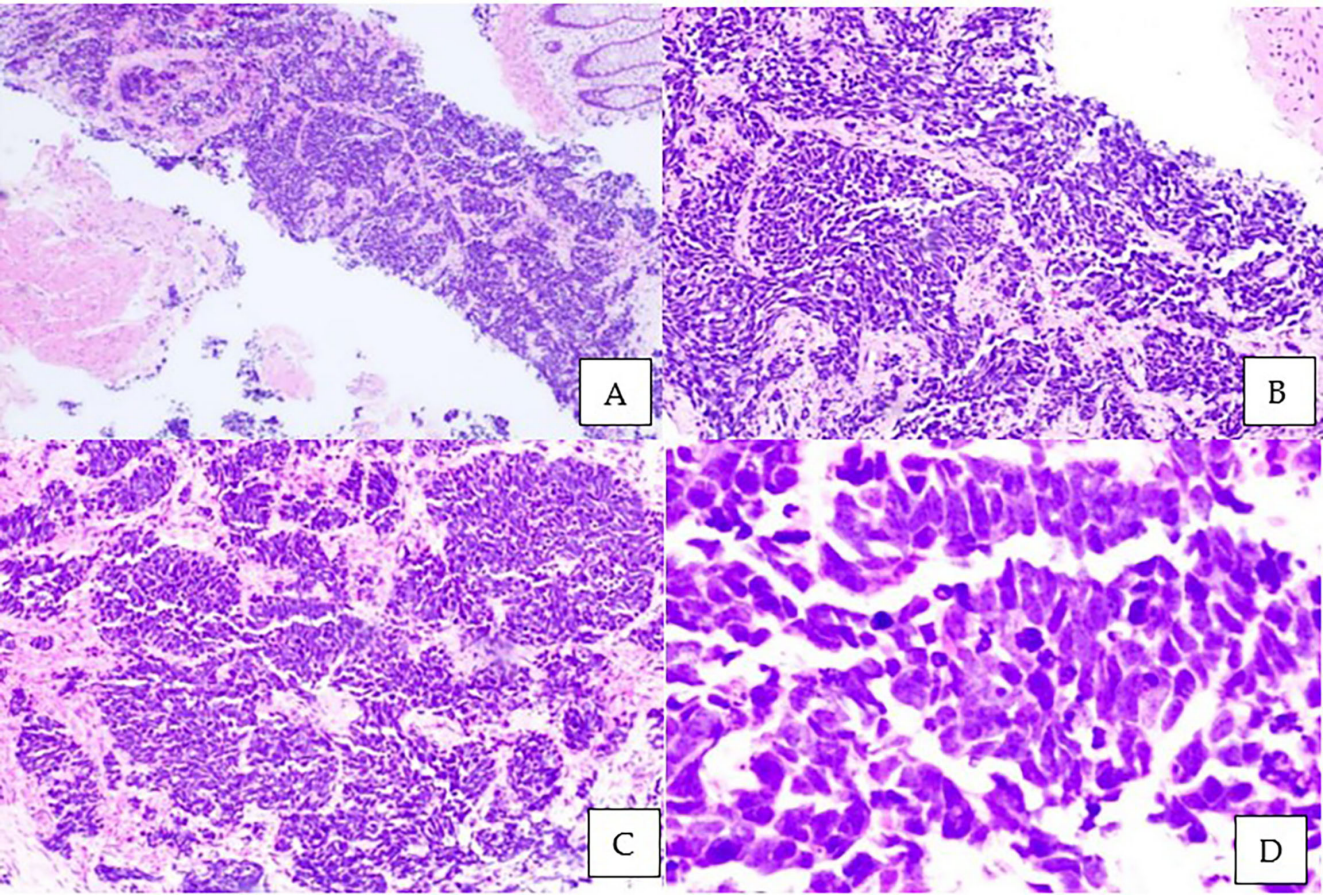
Microscopic examination of the prostate biopsy. Sheets and nests of malignant small ovoid cells with intervening stroma and rectal mucosa floating in the adjacent area. Tumor cells have minimal cytoplasm, hyperchromatic mildly pleomorphic nuclei with stippled chromatin, nuclear molding with peripheral palisading, brisk mitotic activity, apoptotic cells, and occasional necrosis. No glandular or tubule formation and no obvious vascular invasion. **(A)** Hematoxylin and eosin staining (H&E), ×40; **(B)** H&E, ×100; **(C)** H&E, ×200; **(D)** H&E ×400.

**Figure 2:**
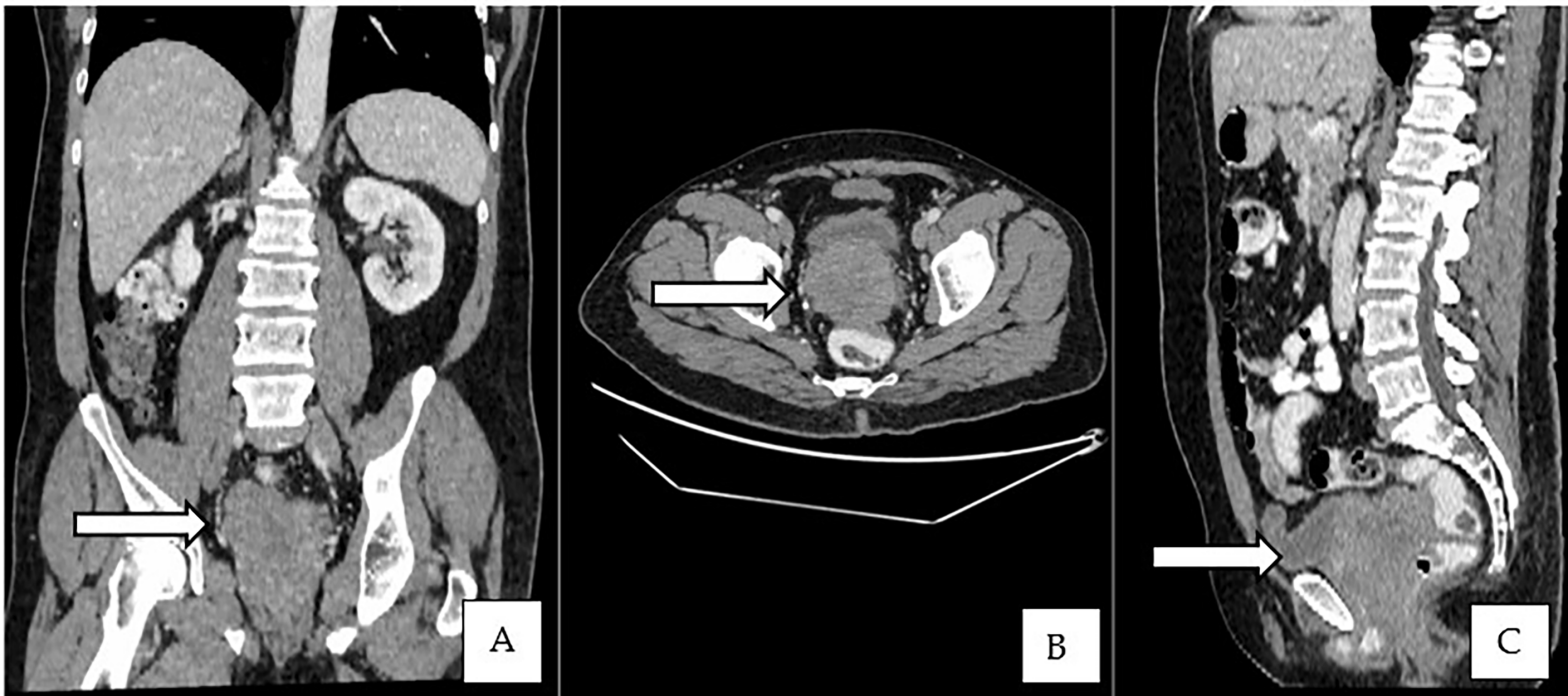
**(A–C)** Contrast-enhanced CT of the abdomen showed an enlarged prostate gland with a lobulated appearance.

**Figure 3:**
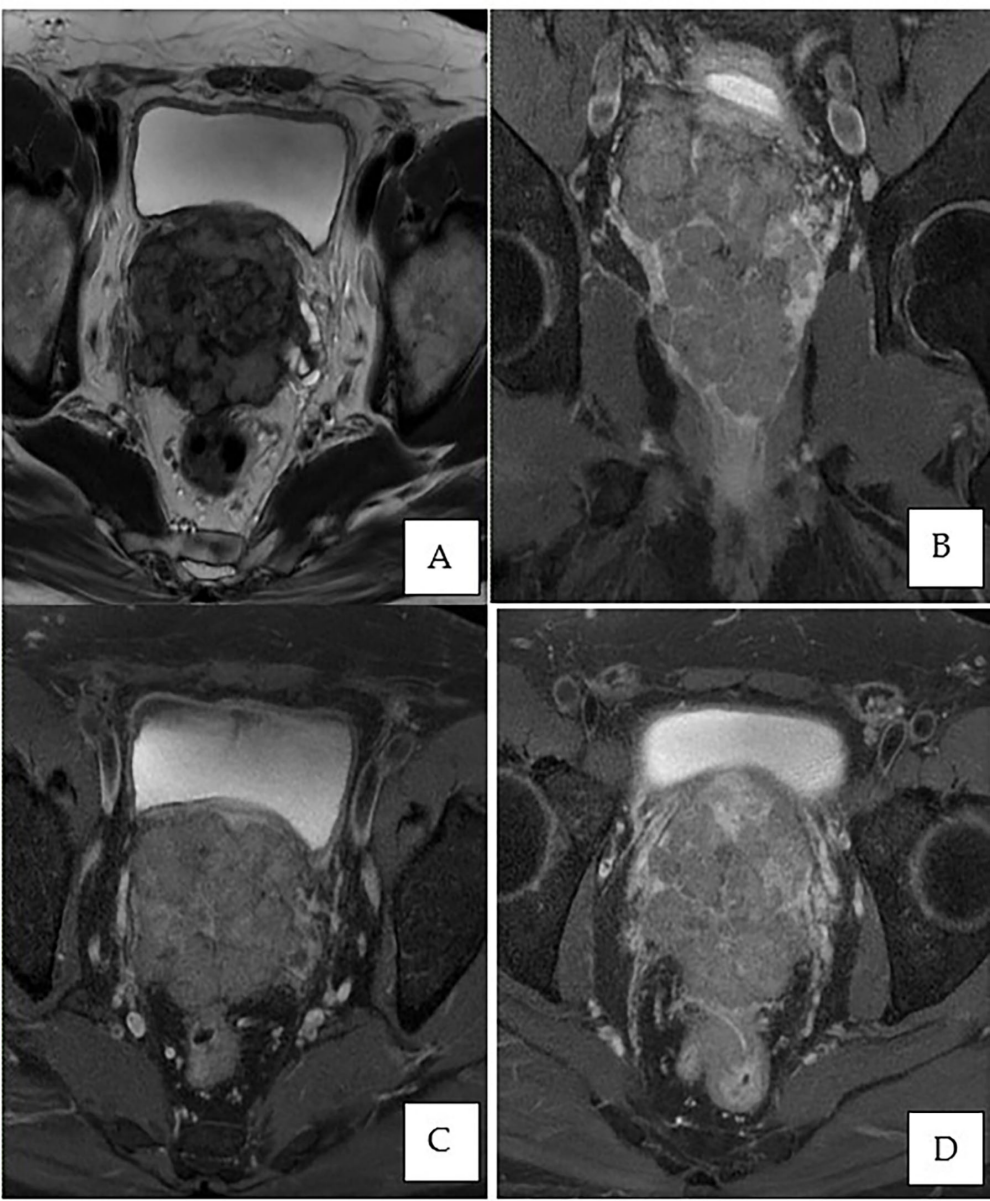
MRI of the prostate. **(A)** Axial T2-weighted image showing an enlarged prostate gland with a multilobulated appearance involving all zones, predominantly the peripheral zone, measuring 7.3 × 7.4 × 9.1 cm (volume: 255 cm^3^). **(B)** Post-contrast coronal T1-weighted fat saturation demonstrates peripheral enhancement extending to the capsule of the prostate. **(C, D)** Post-contrast axial T1-weighted fat saturation exhibiting involvement of the bilateral seminal vesicles and invasion of the rectum.

**Figure 4:**
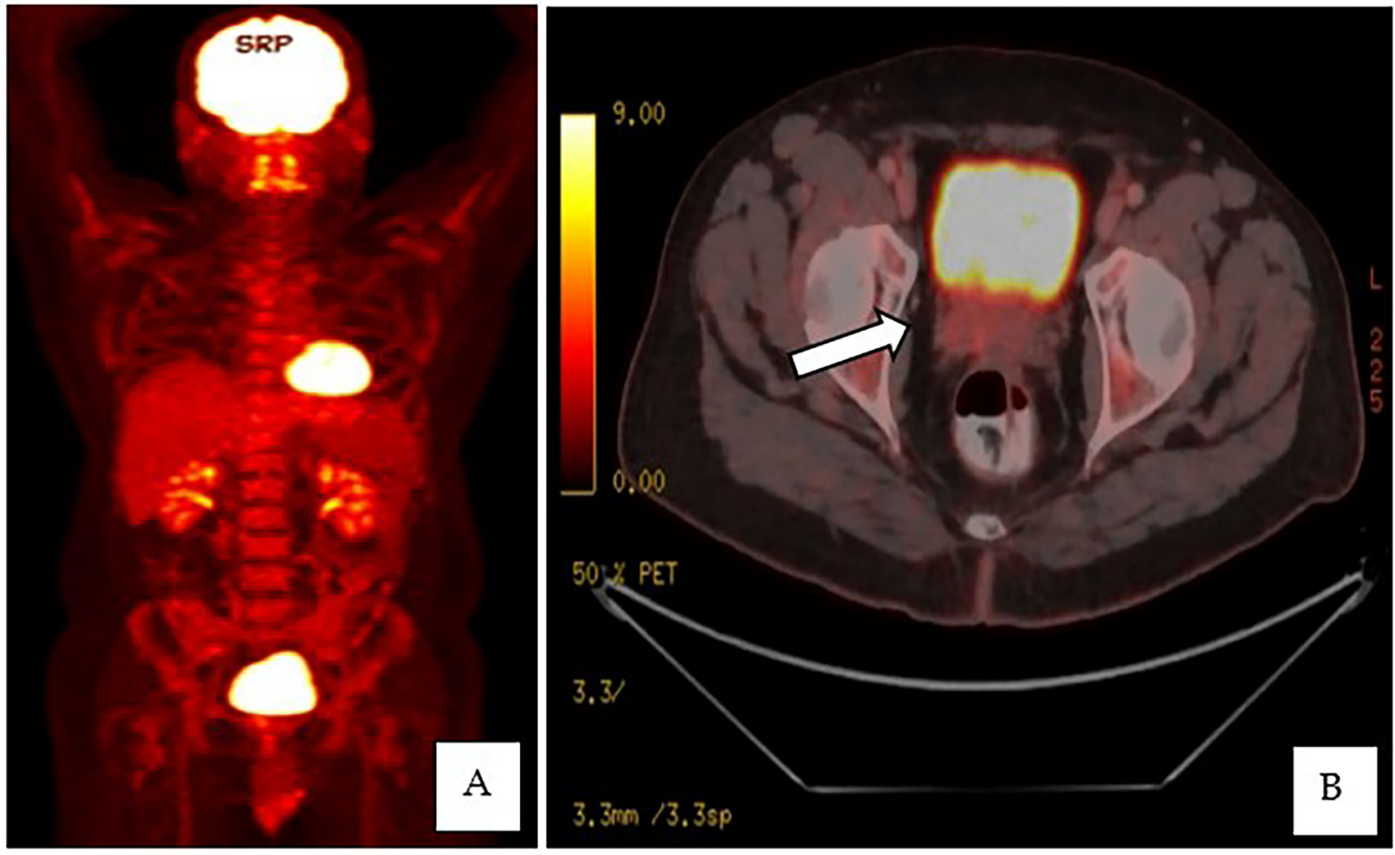
**(A, B)** PET-CT whole-body scan showed an enlarged prostate, measuring 3.5 × 5.6 × 5.1 cm, with an ill-defined hypermetabolic lesion at the right side base of the prostate.

**Figure 5:**
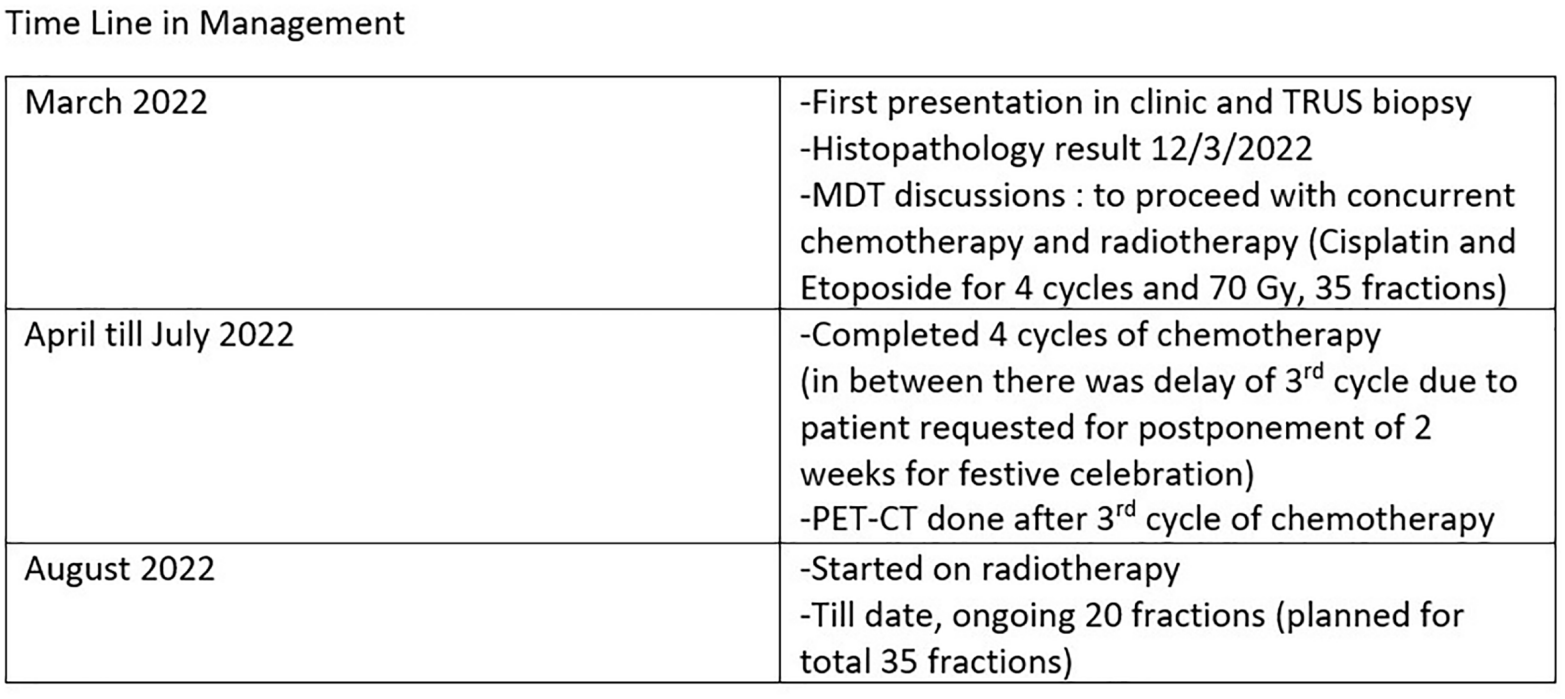
Table showing the management timeline.

This is a 52-year-old Iranian man, with no known medical illness or family history of malignancy. He was not a carrier of any pathogenic variant. He presented with recurrent gross hematuria and perineal pain for 6 months. He did not have any constitutional or other complaints. A digital rectal examination found a huge and hard prostate mass. The prostate-specific antigen (PSA) was 0.8 ng/ml. He underwent a transrectal ultrasound-guided (TRUS) biopsy of the prostate. Histopathology results showed high-grade small cell neuroendocrine carcinoma. Immunohistochemical markers were positive for synaptophysin with a Ki67 index of almost 100%. CD56 and chromogranin A markers were negative (**Figures 1A–D**). Contrast-enhanced computed tomography (CT) scan of the abdomen showed an enlarged prostate gland with a lobulated appearance (**Figures 2A–C**) which was confirmed on multiparametric magnetic resonance imaging (mpMRI). mpMRI of the prostate showed a multilobulated prostatic mass involving all zones, measuring 255 cm³, with invasion to the rectum and bilateral seminal vesicles (**Figures 3A–D**), while CT thorax–abdomen–pelvis ruled out distant metastasis. A multidisciplinary team discussion was held, and the patient was diagnosed with locally advanced prostate neuroendocrine cancer (cT4) and decided for concurrent chemotherapy (cisplatin and etoposide for 4 cycles) and radiotherapy (70 Gy, 35 fractions). He underwent restaging positron emission tomography (PET-CT) whole-body scan after 3 cycles of etoposide and cisplatin and had shown a smaller-size prostate (measuring 50 cm³) compared to that before chemotherapy with a hypermetabolic lesion at the right base of the prostate without evidence of distant metastasis (**Figure 4**). The patient had completed 4 cycles of chemotherapy and an ongoing 20 fractions of radiotherapy to date (**Figure 5**). He also developed right eye blurring of vision and giddiness after the fourth cycle of chemotherapy. A CT brain plain and contrast was done, but there was no focal enhancing lesion. He then underwent a brain MRI scan which showed prominent peri-optic subarachnoid space bilaterally which was consistent with intracranial hypertension without any intracranial lesion. A lumbar puncture, which was unremarkable, was done by a neurology team. The patient was compliant with the therapies, and at the recent follow-up, the patient was well and able to carry out daily activities.

## Discussion

Primary SCPC is an extremely rare form of extrapulmonary neuroendocrine carcinoma, consisting of approximately 0.5% to 1% of all prostate carcinomas. It was first described by Wenk et al. in 1977. It shows an aggressive clinical course and has a poor prognosis. Most of the patients (75%) are diagnosed in advanced stages and are usually unresectable. The median survival is about 2 years. The common sites of metastasis are the lungs, urinary bladder, liver, and bone (7, 13). The Prostate Cancer Foundation proposed a classification for prostate carcinoma with neuroendocrine differentiation, consisting of usual prostate adenocarcinoma with neuroendocrine differentiation, adenocarcinoma with Paneth cell neuroendocrine differentiation, carcinoid tumor, small cell carcinoma, large cell carcinoma, and mixed types [7, 9].

Patients usually presented with complaints related to an enlarged prostate, such as lower urinary tract symptoms (LUTS), most commonly a change in the stream of urination (14, 15). The gold standard of diagnosis is through biopsy. The histology may show a high nuclear/cytoplasmic ratio, nuclear molding, increased mitotic figures, and necrosis. Immunohistochemical markers are the mainstay in diagnosing. The most common markers are chromogranin A, CD56, synaptophysin, and neuron-specific enolase, with synaptophysin being the most sensitive and chromogranin A being the most specific marker. Elevated expression levels of chromogranin A are associated with higher disease burden and poorer prognosis of prostatic carcinoma. Neuroendocrine tumors lack the expression of androgen receptors and do not secrete PSA. Serum PSA does not correlate with disease burden (8, 12, 16, 17).

There are current studies on non-invasive investigations in diagnosing prostatic neuroendocrine tumors. Many serum biomarkers such as antibodies to chromogranin A, synaptophysin, neuron-specific enolase, and CD56 are being used as screening tools for prognostic purposes (17). According to Grigore et al., serum chromogranin A is the most reliable serum marker (8).

Imaging techniques such as PET/CT and metabolic MRI are being proposed as alternatives to prostatic biopsy in diagnosing neuroendocrine tumors. CT typically plays a minimal role in the detection of SCPC and is not recommended for diagnosis. The only role of CT is to show an irregularly enlarged prostate and for nodal staging. CT plays a primary role in metastatic staging for detection and restaging of bone and lung metastases in these cases (18).

While mpMRI is now considered the technique of choice for initial and local (T) tumor staging, PET/CT and PET/MRI have shown great value in distant extraprostatic (N and M) staging. mpMRI can also differentiate prostatic carcinoid from the usual prostatic adenocarcinoma based on the considerably larger size and mild hyperintensity of the tumor on T2W images (18). Recently, biopsy guided by the fusion of MRI and transrectal ultrasound images (called MRI-TRUS fusion biopsy) is increasingly used where MRI findings are used as a reference for US-guided biopsy, allowing for increased accuracy and precision which is frequently done in our center and in this patient as well (18).

Somatostatin receptor (SSR) analogs labeled with positron emitter gallium-68 (^68^Ga) have been developed for PET imaging such as DOTATE which has offered a much higher sensitivity in diagnosing a neuroendocrine tumor as compared to planar and single-photon emission computed tomography (SPECT) imaging. ¹^77^Lu-DOTATE is also being used as a therapeutic agent in neuroendocrine tumors (19, 20). However, these agents are still not widely available in our country.

There is a lack of consensus on the therapy for neuroendocrine tumors, unlike adenocarcinoma in which the therapy is mainly androgen deprivation therapy. For localized disease, prostatectomy can be considered after neoadjuvant chemotherapy (12). However, most cases are presented in advanced stages in which chemotherapy is the main mode of therapy and is related to the treatment of small cell carcinoma of the lung, mainly using platinum-based regimens. Cisplatin is commonly used (7) and it can be used as a single agent or in combination with etoposide or docetaxel as recommended by the National Comprehensive Cancer Network (NCCN) (2, 7, 11). Carboplatin is an alternative single agent used, and concurrent therapy with radiation is commonly used as well. The overall survival is 9 to 13 months. Half of the patients with prostatic neuroendocrine tumors have mixed type, partly adenocarcinoma, and hormonal therapy is also being used in those cases. Neuroendocrine tumors can develop as recurrent tumors in patients receiving hormonal therapy for prostatic adenocarcinoma. The other types of neuroendocrine tumors are large cell carcinoma and carcinoid tumor, which are extremely rare.

Recent studies propose prostate neuroendocrine tumor arising from a “tumor cell autonomous” phase, in which the tumor is enriched with androgen-negative cells. The main characteristic is the loss or mutation of tumor-suppressor genes like *RB* and *p53*, leading to genetic instability and, thus, affecting cell cycle genes, especially in M-phase transition, including aurora kinase A (*AURKA*) and proline kinase 1 (*PLK1*). According to Beltran et al., MYCN amplification in tumor cells leads to a neuroendocrine property, contributing to a better understanding of the molecular basis of neuroendocrine tumors, and targeted therapy, for example, using *AURKA* inhibitors such as danusertib, has been proposed in clinical trials (21).

The current challenge being faced is the lack of a definite study demarcating the difference between metastasis from a distant site such as the lungs or a local site like the bladder and primary neuroendocrine carcinoma.

## Conclusion

In conclusion, we present a case of a locally advanced small cell prostate neuroendocrine carcinoma with rectum invasion without distant metastasis. It is extremely rare to diagnose a case of a locally advanced disease without distant metastasis due to the aggressive nature of the disease. Neuroendocrine prostate cancer should be suspected when there is a low PSA level with a prostate mass on examination. It is diagnosed through clinical and radiological examination with final confirmation from the histopathology result. TRUS biopsy, multiparametric MRI, and MRI-TRUS fusion biopsy have increasing roles in imaging assessment and disease management.

It is important to involve multidisciplinary teams in the management of the disease; however, there is still a lack of consensus on management due to the rarity of the disease. The main modality of management in advanced disease (stage IV) is chemotherapy, which is based on small cell lung carcinoma therapy. It is highly aggressive with a poor prognosis and is not responsive to hormonal therapy. To date, targeted therapy, for example, *AURKA* inhibitors such as danusertib, has been proposed in clinical trials.

## Limitation

Neuroendocrine prostate cancer, especially pure small cell prostate cancer, is a rare disease. The recent literature available mainly includes case reports and case series. The current challenge being faced is the lack of prospective studies, and standard treatments are based on therapy of small cell lung cancer.

## Data availability statement

The original contributions presented in the study are included in the article/supplementary material. Further inquiries can be directed to the corresponding author.

## Ethics statement

Written informed consent was obtained from the patient for the publication of his case and associated images.

## Author contributions

ST prepared the draft. FI conceptualized the article and edited and finalized the draft. IR prepared the images, photos, and captions, and edited the draft. WW prepared the slides. All authors contributed to the article and approved the submitted version.

## Funding

The publication fees will be borne by our institution.

## Acknowledgments

The authors would like to express their deepest appreciation to all the parties who have contributed in managing the patient and completing the case report.

## Conflict of interest

The authors declare that the research was conducted in the absence of any commercial or financial relationships that could be construed as a potential conflict of interest.

## Publisher’s note

All claims expressed in this article are solely those of the authors and do not necessarily represent those of their affiliated organizations, or those of the publisher, the editors and the reviewers. Any product that may be evaluated in this article, or claim that may be made by its manufacturer, is not guaranteed or endorsed by the publisher.
